# New frontiers in immune checkpoint B7-H3 (CD276) research and drug development

**DOI:** 10.1186/s12943-023-01751-9

**Published:** 2023-03-02

**Authors:** Ayechew Adera Getu, Abiye Tigabu, Ming Zhou, Jianrong Lu, Øystein Fodstad, Ming Tan

**Affiliations:** 1 Institute of Biochemistry and Molecular Biology, Institute of Biomedical Sciences, and Research Center for Cancer Biology, China Medical University, Taichung, Taiwan; 2 Department of Physiology, School of Medicine, College of Medicine and Health Sciences, University of Gondar, Gondar, Ethiopia; 3 Cancer Research Institute and School of Basic Medical Sciences, Central South University, Changsha, China; 4 Department of Biochemistry and Molecular Biology, College of Medicine, University of Florida, Gainesville, USA; 5 Department of Tumor Biology, Institute for Cancer Research, Oslo University Hospital Radiumhospitalet, Oslo, Norway

**Keywords:** B7-H3, CD276, Cancer, Immunotherapy, Drug Development

## Abstract

B7-H3 (CD276), a member of the B7 family of proteins, is a key player in cancer progression. This immune checkpoint molecule is selectively expressed in both tumor cells and immune cells within the tumor microenvironment. In addition to its immune checkpoint function, B7-H3 has been linked to tumor cell proliferation, metastasis, and therapeutic resistance. Furthermore, its drastic difference in protein expression levels between normal and tumor tissues suggests that targeting B7-H3 with drugs would lead to cancer-specific toxicity, minimizing harm to healthy cells. These properties make B7-H3 a promising target for cancer therapy.

Recently, important advances in B7-H3 research and drug development have been reported, and these new findings, including its involvement in cellular metabolic reprograming, cancer stem cell enrichment, senescence and obesity, have expanded our knowledge and understanding of this molecule, which is important in guiding future strategies for targeting B7-H3. In this review, we briefly discuss the biology and function of B7-H3 in cancer development. We emphasize more on the latest findings and their underlying mechanisms to reflect the new advances in B7-H3 research. In addition, we discuss the new improvements of B-H3 inhibitors in cancer drug development.

## Introduction

Supporting the immune system to kill cancer cells is a promising treatment strategy in cancer therapy. Immune checkpoints (e.g. PD-1, PD-L1, CTL4) regulate the immune system critical for self-tolerance, preventing autoimmunity, and fighting invading cancer cells [1]. Blocking elements of this checkpoint system has changed the paradigm of cancer therapy and achieved significant success in patient survival [2, 3].

B7-H3 (also known as CD276), a member of the B7 family of immune checkpoint proteins, is highly expressed in cancer cells and activated tumor-infiltrating immune cells, and helps cancer cells to evade the surveillance of cytotoxic T-cells and natural killer cells [4]. Emerging evidence have shown that B7-H3 is involved in tumor proliferation, metastasis, treatment resistance, resulting in poor patient outcome, and hence B7-H3 has emerged as a promising therapeutic target for anti-cancer therapy [5, 6].

Extensive research on B7-H3 has been carried out in the past decade, broadening our understanding of this molecule. In recent several years, new findings on B7-H3 functions and its multifaceted roles in cancer development have been obtained. Here, we provide an overview of the current understanding about B7-H3 and its involvement in the pathogenesis of cancer and potential functions in other health conditions. We put an emphasis on the new developments, including but not limited to the impact of B7-H3 on cancer cell metabolism, senescence, obesity, link to microbiota, cancer cell stemness and posttranslational modification of B7-H3. We also discuss the current trends in drug developments in targeting B7-H3.

## The biology of B7-H3

### B7 family proteins

The B7 family proteins are a type of integral membrane proteins found on activated antigen-presenting cells and consists of structurally related cell-surface protein ligands that bind to receptors on lymphocytes [7]. B7.1 (CD80) and B7.2 (CD86) are the two major types of B7 proteins, but currently, there are other proteins grouped in the B7 family, including inducible co-stimulator ligand (ICOS-L), and co-inhibitory programmed death-1 ligand (PD-L1), programmed death-2 ligand (PD-L2), B7-H3, and B7-H4 (Table 1).

**Table 1:** B7 family proteins and their binding partners

B7 family ligands	Alternative names	Receptor (binding) partners	Effect of ligand-receptor interaction	References
B7-1	CD80	CD28, CTLA-4, PD-L1	Co-stimulatory or Co-inhibitory	https://doi.org/10.1073/pnas.0507257102
B7-2	CD86	CD28, CTLA-4	Co-stimulatory or Co-inhibitory	https://doi.org/10.1073/pnas.0507257102
B7-DC	PD-L2, CD273	??, PD-1	Co-stimulatory or Co-inhibitory	https://doi.org/10.1084/jem.20050072
B7-H1	PD-L1, CD274	??, PD-1	Co-stimulatory or Co-inhibitory	https://doi.org/10.1038/70932, https://doi.org/10.1016/j.hemonc.2013.09.005
B7-H2	B7RP1, CD275	ICOS	Co-stimulatory	PMID: 21,530,327
B7-H3	CD276	??	Co-stimulatory or Co-inhibitory	https://doi.org/10.1038/85339, https://doi.org/10.1038/ni967,
B7-H4	VTCN1, B7x, B7S1	??, BTLA	Co-stimulatory or Co-inhibitory	https://doi.org/10.1016/S1074-7613(03)00152-3
B7-H5	VISTA, Platelet receptor Gi24, SISP1	??	Co-inhibitory	https://doi.org/10.1084/jem.20100619
B7-H6	NCR3LG1	NKp30	Co-stimulatory effect for NK cells	https://doi.org/10.1007/s00251-012-0616-2
B7-H7	HHLA2	CD28H	Co-stimulatory or Co-inhibitory	https://doi.org/10.1073/pnas.1303524110

The B7 family produces a costimulatory or a coinhibitory signal to enhance or decrease the activity of the MHC-TCR signal between the antigen presenting cells (APC) and the T cells. Interaction of B7-family members with costimulatory receptors augments immune responses while interaction with coinhibitory receptors attenuates immune responses [8, 9].

B7-H3 shares 20–27% amino acid identity with other B7 family members [10]. It is a type-I transmembrane protein that primarily functions as a negative immunoregulatory protein, and is overexpressed in various human tumor tissues [4–6, 11].

### Structure of B7-H3

**Fig. 1:**
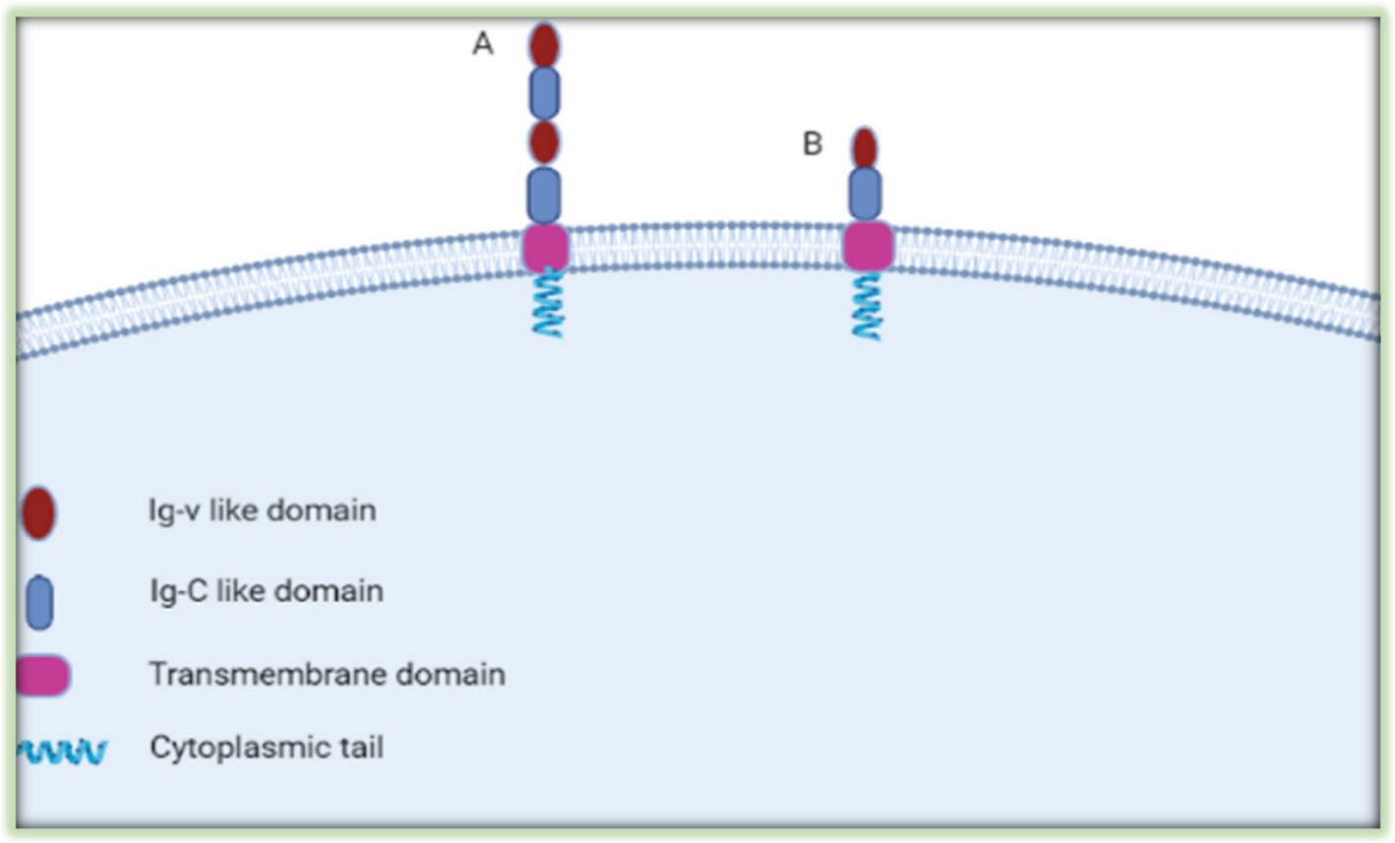
Structure of B7-H3 Protein. The dominant form of human B7-H3 is 4IgB7-H3. It includes two identical pairs of IgV-like and IgC-like domains (**A**), and mouse B7-H3 is 2IgB7-H3, it includes a single pair of IgV-like and IgC-like domains (**B**)

The basic structure (2Ig form) of B7-H3 contains a single pair of IgV-like and IgC-like immunoglobulin domains, a transmembrane region, and a short highly diverse cytoplasmic tail [12] (Fig. 1). The dominantly expressed form of human 4IgB7-H3 contains tandemly duplicated VC domains with four Ig-like domains [13]. Although human B7-H3 has two isoforms (2IgB7-H3 and 4IgB7-H3), the mouse B7-H3 has only one isoform (2IgB7-H3) [14]. Serine and arginine-rich splicing factor 3 (SRSF3) involves the splicing of B7-H3 by directly binding to its exon 4 and/or 6 [15]. B7-H3 crystallized as an unusual dimer arising from the exchange of the G strands in the IgV domains of partner molecules, which indicates the dynamic nature and plasticity of the immunoglobulin fold [16]

### The Cellular Localization of B7-H3

B7-H3 has been observed to be expressed in different cellular compartments and different cancer types may have different B7-H3 localization profiles. Several immunostaining results show B7-H3 was expressed on the cell membrane and in cytoplasm of tumor tissues [17–19]. Zanjani S et al. showed a higher cytoplasmic expression of B7-H3 than that of the membranous expression in clear cell renal cell carcinoma (ccRCC) [20]. B7-H3 has been reported to be expressed in the nucleus of 30% of colon cancer and the expression of nuclear B7-H3 was associated with poor overall survival. In addition, B7-H3 expression was detected in tumor-associated vasculature and fibroblasts of most colorectal cancer samples [21]. Confocal microscopy of fibroblast-like synoviocytes (FLS) and T cell co-cultures showed localization of B7-H3 in the region of the T cell-FLS contact point [22]. B7-H3 is expressed mainly in the stromal compartment of gastric cancer [23] and it induces exosome secretion. Intracellular upregulation of B7-H3 increases the presence of B7-H3 in exosomes secreted from cancer cells [24].

### The distribution and expression of B7-H3 in normal tissues and cancer

B7-H3 is overexpressed in tumor tissues while its expression is low in normal tissues [25]. B7-H3 overexpression and its negative correlation with patient survival has been reported in various malignancies [17, 26–41]. Additionally, B7-H3 is expressed in immune cells; monocytes, dendritic cells, myeloid derived suppresser cells (MDSCs), neutrophils, macrophages, B cells, and activated T cells. Furthermore, B7-H3 is also expressed in normal tissues and body fluids at very low levels, including epithelial cells, pleural effusion, anterior pituitary progenitor cells and human serum [8, 11, 42, 43].

**Fig. 2:**
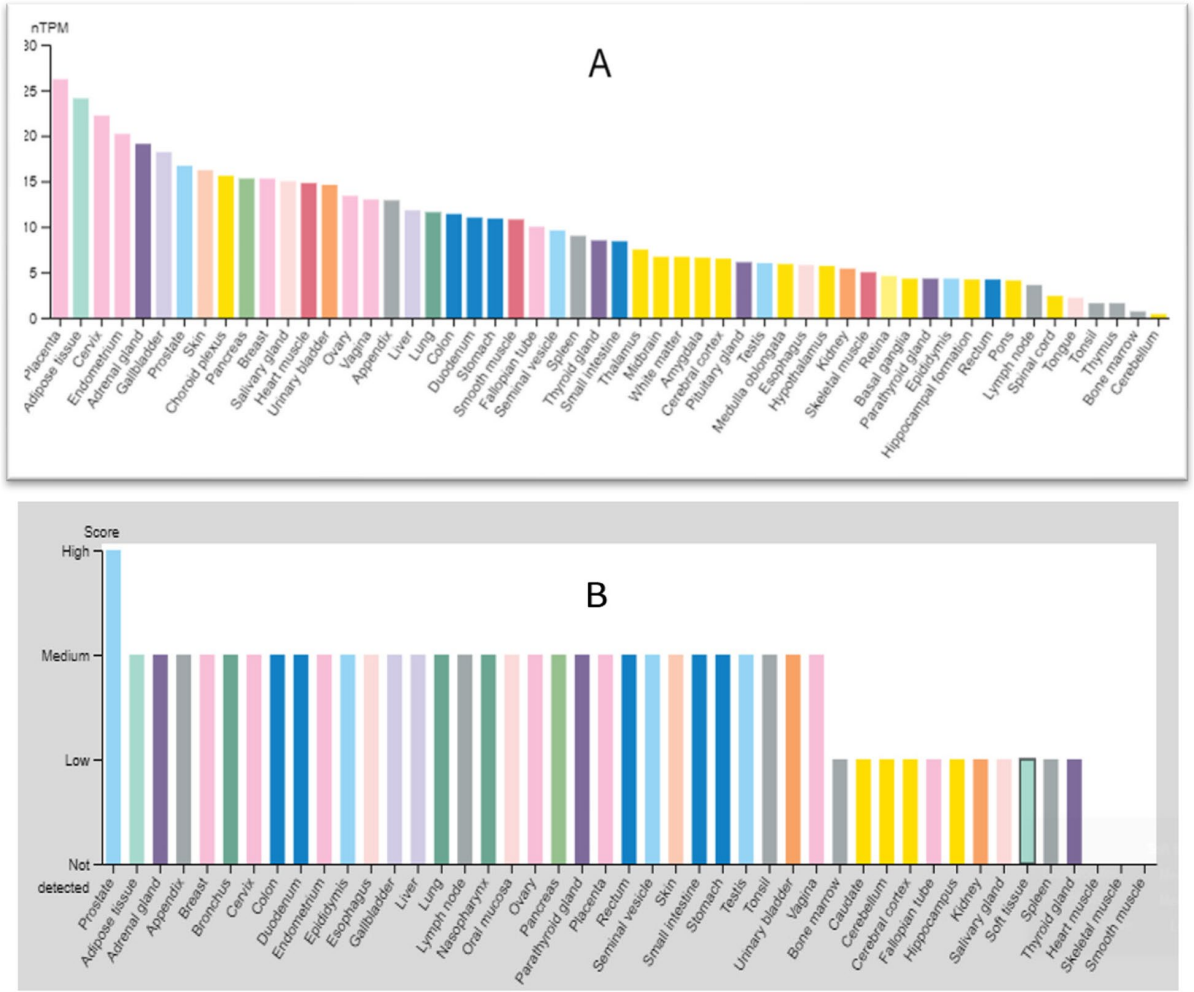
Human B7-H3 expression levels in different tissues. Human B7-H3 mRNA expression(**A**) and protein expression(**B**) level across human tissues (Source: https://www.proteinatlas.org)

As shown in the Fig. 2, the human B7-H3 mRNA and protein expression levels were analyzed across human tissues using RNA sequencing datasets from the Genotype-Tissue Expression (GTEx), FANTOM5, and The Human Protein Atlas (HPA) project. Combining all three sources showed that B7-H3 mRNA expression level is highest in placental tissue and lowest in cerebellum tissue (Fig. 2A), whereas protein expression of B7-H3, although low compared to tumor tissues, is highest in normal prostate tissue but almost not detectable in muscle tissues (Fig. 2B).

### Regulation of B7-H3 expression

Differential expression of B7-H3 at protein level suggests that the post-transcriptional regulation is critical for its expression. To this end, different miRNAs bind directly or indirectly to the B7-H3 RNA and regulate its protein expression levels in cancer cells [44]. MicroRNA-199a, miR-128, and microRNA-187 regulate the expression of B7-H3 in different cancers via direct binding to the 3″ UTR of B7-H3 mRNA. In addition, BRD4, ILT-4 and ELK1 regulate B7-H3 expression through PI3K/AKT/mTOR signaling and impact B7-H3 at the transcriptional or epigenetic level [45–50]. Zhao S et al. also identified that SUPT20H (SP20H) negatively regulates B7-H3 expression while eIF4E positively regulates B7-H3 expression in various cancer cells. Activation of p38 MAPK-eIF4E signaling axis serves as a key regulator of transcription initiation and protein expression of B7-H3 in tumor cells [51]. Recently, B7-H3 gene promotor was found to be hypomethylated in ankylosing spondylitis patients, whereas B7-H3 expression was significantly elevated, suggesting that B7-H3 gene is under epigenetic control [52].

## B7-H3 and cancer pathogenesis

### B7-H3 and cancer patient outcome

The temporal and spatial overexpression of B7-H3 in a variety of cancers but low in normal tissues indicated the pathological significance of this immune regulatory protein. Its degree of expression and localization in the blood [53–55], cerebrospinal fluid(CSF) [56], in exosomes [57, 58], and nucleus [21] has been associated with clinicopathologic features of cancer and patient survival. Crispen et al. reported that enhanced tumor expression of B7-H3 correlates with adverse clinical and pathologic features of clear cell renal cell carcinoma and independently predicts disease progression and cancer-specific death [59].

Moreover, higher expression of B7-H3 is more frequently observed in patients with metastatic cancer than in those with localized cancer. For example, patients with metastatic prostate cancer, high B7-H3 expression was independently associated with high disease-specific mortality and overall mortality rates [34]. This indicates that B7-H3 is involved in cancer metastasis capacity.

In fact, high levels of B7-H3 expression have been found in all the cancer types tested. In a meta-analysis study of 24 observational studies consisting of 4,141 patients, an elevated baseline B7-H3 did significantly correlate with poor overall survival (OS) and recurrence free survival (RFS) across a wide range of tumor types [16]. The study concludes that elevated B7-H3 expression is significantly associated with poor survival in cancer patients. Therefore, although certain earlier studies [60–62] reported opposing and inconclusive results about B7-H3 in cancer, numerous studies have demonstrated that the high expression of B7-H3 in various human cancer types correlates with poor patient outcomes, and this molecule has emerged as a promising target for cancer therapy.

### Immunologic functions of B7-H3

**Fig. 3:**
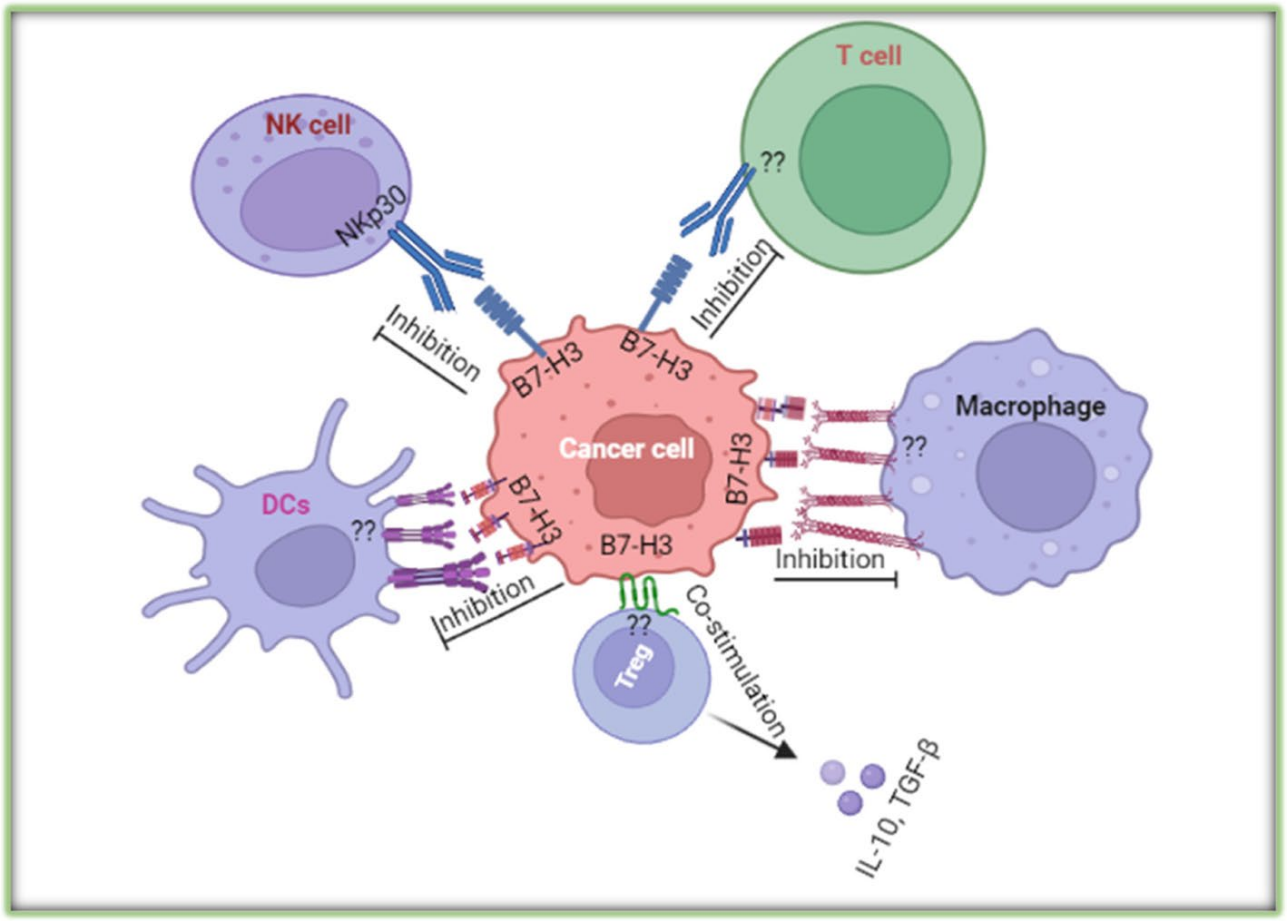
A diagram of the interaction of cancer cell expressed immune checkpoint B7-H3 with immune cells

B7-H3 influences immune responses and cancer progression through immunological and non-immunological pathways (64–66). Although the receptor for B7-H3 has not been identified, it is assumed that the activated CD4^+^ and CD8^+^ T cells express a receptor that can be recognized by B7-H3 expressed on APC cells or tumor cells [63, 64]. The 2Ig VC and 4Ig VCVC forms of human B7-H3 inhibit T cell proliferation and downregulate cytokine production [16]. Expression of B7-H3 favored an immunosuppressive microenvironment by promoting the production of IL-10, TGF-β1 [65], and inhibiting the activity of CD4^+^ T cells, CD8^+^ T cells, γδT cells, CAR-T cells, Vδ2 T cells, Th17 cells, CD3^+^ T cells, NK cells, macrophages, neutrophils, dendritic cells, and also inhibiting IFN-γ, IL-2, perforin, granzyme B secretion [66–69] (Fig. 3).

B7‑H3 regulates the differentiation of tumor‑associated macrophages and promotes the polarization of type 2 macrophages and switching M1 phenotype to the M2 phenotype [70], and B7-H3 also contributes to CCL2–CCR2–M2 macrophage axis-mediated immunosuppression [71]. Moreover, tumor-derived granulocyte macrophage colony stimulating factor (GM-CSF) activates neutrophils and induces neutrophil B7-H3 expression via JAK-STAT3 signaling pathway [72], while miR‑34a induces immunosuppression through modulating a SIRT1/NF‑κB/B7‑H3/TNF‑α axis [73]. Furthermore, FOXP3^+^ regulatory T cells positively associated with B7-H3 expression and resulting an immunosuppressive tumor microenvironment [74]. However, some studies reported that patients with high tumoral B7-H3 expression showed increased numbers of immune cells; CD8^+^ T-cells, CD4^+^ T cells, natural killer cells, plasmacytoid dendritic cells and interferon-γ production [62, 75].

### Non-immune regulatory functions of B7-H3

#### B7-H3 and tumor proliferation

**Fig. 4:**
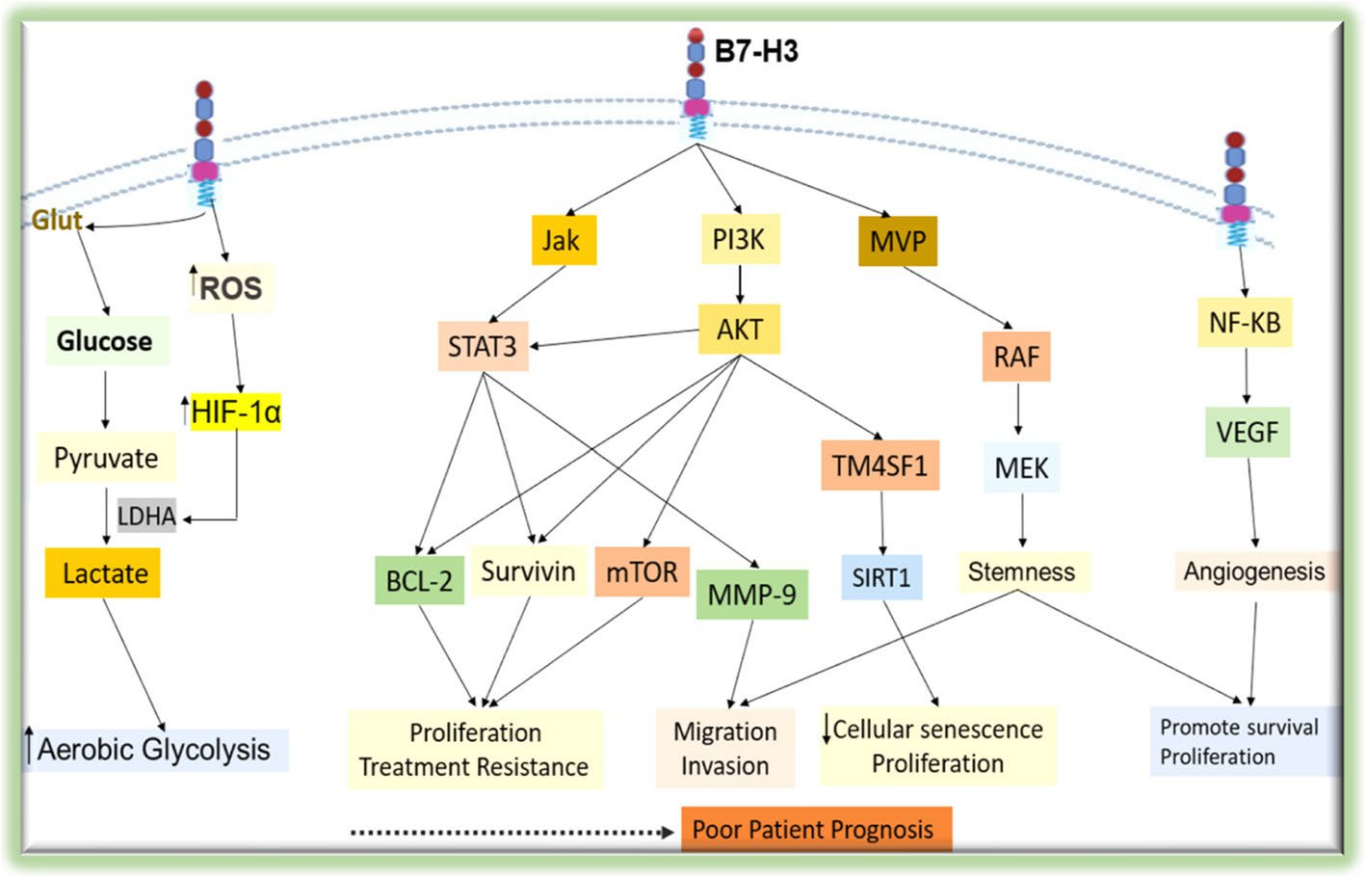
Summary of the molecular mechanisms of the tumorigenic effects of B7-H3. B7-H3 expressed on cell membrane triggers different signaling cascades to activate downstream molecules that contribute to the malignant behaviors of cancer cells

Cancer is heterogeneous and complex diseases characterized by the development of abnormal cells that divide uncontrollably and can infiltrate and invade normal tissues. The capacity of cancer cells to grow and proliferate is determined by the tumor microenvironment and the cancer cell itself [76]. The role of B7-H3 in the proliferation of cancer cells has been documented in cervical cancer [54], gastric cancer [77] and many other cancer types [78, 79]; whereas in a few studies significant proliferative effect was not observed [80, 81]. In addition to regulating the immunological microenvironment, B7-H3 has been reported to activate signaling pathways such as ERK, PI3K, and Stat3 in cancer cells, which may lead to the accelerated cell proliferation and tumor growth [82, 83] (Fig. 4).

#### B7-H3 Involvement in angiogenesis and cancer cell metabolism

Angiogenesis is a hallmark of cancer and is intricate in the tumorigenesis of solid tumors. As tumors proliferate and grow, the oxygen availability in the tumor microenvironment decreases and leads to tumor hypoxia. Tumors develop adaptive mechanisms to sustain their growth by activation of hypoxia-inducible factor(HIF) [84], which is the master regulator of the angiogenesis promoting program. Tumor cells can overcome the hypoxic environment by inducing angiogenesis, through the expression of vascular endothelial growth factor (VEGF) [79], a transcriptional target of HIF. B7-H3 is shown to be implicated in angiogenesis in a variety of cancer diseases [79, 85, 86]. B7-H3 promotes angiogenesis by stimulating the secretion of VEGF [87]. In colorectal cancer, using in-vitro and in-vivo experiments, overexpression of B7-H3 promoted tumor angiogenesis by upregulating VEGF expression via activating the NF-κB pathway [79].

The rapidly proliferating cancer cells need metabolic reprogramming to maintain their energy supply. The pathways for nutrient acquisition and preference, synthesis of biomolecules and bioenergetics are reprogrammed such that tumor cells’ proliferation capacity and survival is maintained [88]. Beyond the immune regulation, the B7-H3 protein has been appreciated in non-immune regulatory functions including energy metabolism. B7-H3 is implicated in the regulation of glucose uptake and metabolism in tumor cells. Lim et al. reported that B7-H3 increased the uptake of glucose and lactate production [89] which supports the concept of Warburg effect [90].

It has been shown that B7-H3 increases the reactive oxygen species (ROS) production through an unknown mechanism in the cytosol of tumor cells and promotes the stabilization of HIF-1α to increase glycolysis [89]. In consistence with this finding, in the oral squamous cell carcinoma cells, B7-H3 enhanced glycolysis through the upregulation of HIF-1α and its downstream targets including Glut1 through PI3K/Akt/mTOR pathway [91]. A decreased expression of B7-H3 has been noted to reduce glycolytic capacity of breast cancer cells [92]. These studies clearly indicate that B7-H3 is involved in cancer metabolic flux and support its pathogenesis.

#### Effect of B7-H3 on tumor treatment resistance

Enhanced expression of B7-H3 in tumors has been linked with therapeutic resistance, metastasis potential and poor patient prognosis [93–95]. The feature of developing treatment resistance poses a huge burden to the cancer patients and is the major factor for poor prognosis.

B7-H3 has been linked to treatment resistance also in cancer cells [92, 96]. Liu et al. showed the effect of B7-H3 on paclitaxel induced cytotoxicity. In their study, breast cancer cell lines were more sensitive to paclitaxel when B7-H3 was silenced which may be associated with the prevention of the activation of the Jak2/Stat3 pathway [97]. In ovarian cancer cell lines, Zhou et al. reported that overexpression of B7-H3 induced the activation the PI3K/AKT signaling pathway and up-regulated BCL-2 in protein level, resulting in the sustained growth and chemo-resistance [98]. These studies demonstrate that B7-H3 plays an important role in inducing therapeutic resistance.

## New advances of B7-H3 research

### B7-H3 and cancer stem cells

Stem cells are defined as cells that have the ability to self-renewal and produce mature cells of a particular tissue through the process of differentiation [99]*.* Self-renewal is the hallmark property of stem cells in normal and neoplastic tissues. However, the existence of cancer stem cells (CSCs) has been a subject of controversy for several years. Nevertheless, researchers have identified CSCs in cancers including leukemia, breast, colon, prostate, brain, ovarian and pancreatic cancers [100]. Scientists argue that understanding self-renewal mechanisms or pathways of CSCs is critical for better characterization of the phenotypical features of these stem cells. For instance, it has been reported that Wnt pathway plays a critical role in the initiation and maintenance of CSCs [101].

A failure to maintain cellular homeostasis through repair or early removal of the cells harboring gene mutation by the immune system would lead to uncontrollable proliferation of cells. CSCs play a central role in implementing immune evasion mechanisms. These cells, through their immunomodulatory strategies, can protect themselves against immune monitoring and eradication [102]. Because of this characteristics*,* CSCs are considered to be responsible for metastasis of the disease, treatment failure, recurrence, and unfavorable patient outcome [103]. It has been documented that traditional chemotherapeutic drugs may only kill cancer cells but spare the CSC population and lead to tumor recurrence [104]. Developing therapeutics targeting pathways so as to minimize the disease recurrence and risk of treatment resistance should be a focus of future studies.

B7-H3 has been found to cause enrichment of CSCs. Liu et al. revealed that B7-H3 in stem cell populations were over- expressed as compared to the bulk of the breast cancer cells. They asserted that over- expression of B7-H3 dramatically increased the cancer stem cell pool size. Furthermore, B7-H3 over- expression enriched the CSCs and contributed to drug resistance [105]. In a prostate cancer cells model, B7-H3 was abundantly expressed in prostate cancer stem cells compared to total tumor cell population [106]. Moreover, in HeLa cervical carcinoma cells, it was found that sphere-forming cells expressed various stem cell markers and that these cells also expressed significant amount of B7-H3 [107], indicating that the expression level of B7-H3 positively correlated with the proliferation, self-renewal, and oncogenesis.

Another significant study performed by Wang, C et al., indicated a higher degree of expression of B7-H3 in CSCs of human head and neck squamous cell carcinoma (HNSCC). Importantly, high expression of B7-H3 helped CSCs escape immune surveillance in HNSCC initiation, progression, and metastasis. Blockage of B7-H3 with monoclonal antibodies eliminated CSCs and inhibited tumor growth and metastasis by enhancing CD8 + T lymphocyte-mediated anti-tumor immunity [108]*.*

Overall, B7-H3, though well documented to be highly expressed in bulk cancer cells, is even higher expressed in CSCs and contributes to the initiation of tumor development, progression, metastasis, and therapeutic resistance.

### B7-H3 and cellular senescence

Senescence is the steady process of stopping cell cycle and cellular growth. This process not only contributes to aging and age-related illnesses but also protects cells from not be cancerous [109]*.* Senescent cells have been observed in diseases such as diabetes, atherosclerosis, diabetes, and cancer [110, 111].

Cellular senescence can be induced by a variety of endogenous and exogenous stress and damage signals which can be accumulated during their lifetime. Telomere shortening, oncogenic stress, nutrient depletion, cancer chemotherapies, and lysosomal or endoplasmic reticulum stress are fairly known as stimuli triggering cellular senescence [112].

Cells of colon cancer, prostate cancer, lymphomas and breast cancer have been observed to show senescence markers during their development [113, 114]. Tumor cell senescence has been appreciated as the homeostatic and protective barrier against tumor initiation and development. Therefore, inducing tumor cell senescence by anticancer interventions may be used as a strategy for cancer treatment [115]. As an immune check point molecule, B7-H3 inhibits cytotoxic immune cells in the tumor microenvironment and contributes to tumor growth. A few studies reported the involvement of B7-H3 in cancer cell senescence. For instance, Lehmann et al. showed that radiation therapy induced senescence was associated with a significantly increased release of exosome-like microvesicles which were enriched with B7-H3 [58].

Wang, R. et al., also reported that higher expression of B7-H3 worsens the resistance to a low-dose doxorubicin-induced senescence in colorectal cancer [116]. They showed that higher expression of B7-H3 prevented cellular senescence and growth arrest through the AKT/TM4SF1/SIRT1 pathway. Importantly, blocking the AKT/TM4SF1/SIRT1 pathway significantly reversed the B7-H3-induced resistance to cellular senescence. These reports indicated a possible involvement of B7-H3 in therapy- induced cancer cell senescence. Therefore, detailed understanding of the role of B7-H3 in cellular senescence is warranted as targeting B7-H3 might be a promising treatment strategy to promote cancer cell senescence.

### B7-H3 and adipose tissue, obesity, and diabetes mellitus

**Fig. 5:**
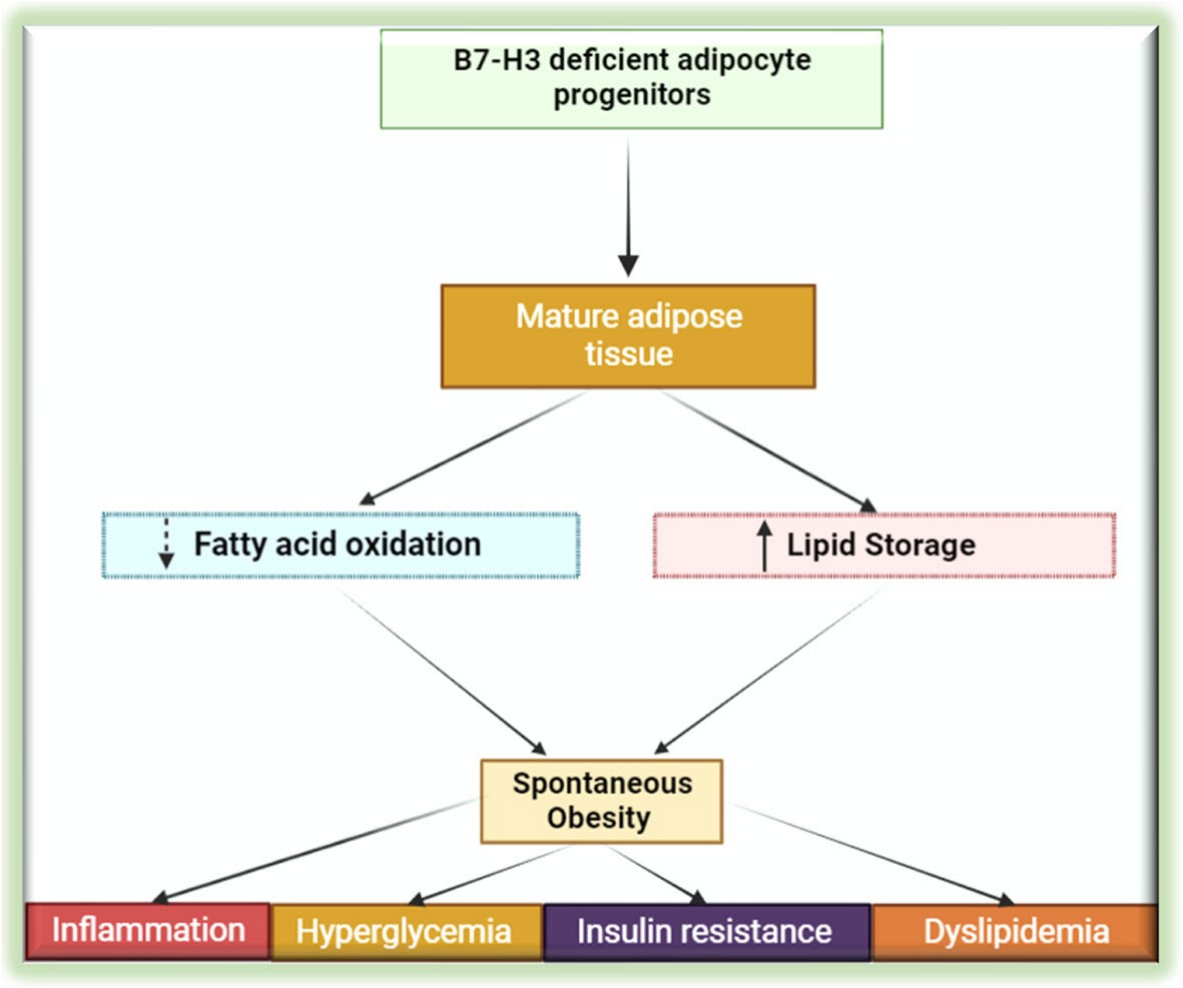
B7-H3 plays an important role in adipocyte progenitor cell differentiation, lipid oxidation, and obesity. Mature adipose cells derived from adipose progenitors lacking B7-H3 stores more fat and lacks of B7-H3, which increases the risk of obesity and metabolic syndrome in the mouse model

Several studies have shown that B7-H3 contributes to the supply of energy for proliferative tumor cells by controlling aerobic glycolysis [117, 118] suggesting its involvement in regulating cellular energy metabolism. The expression of B7-H3 in normal tissues is very low as compared to activated immune cells and cancer cells. However, Picarda et al., (2022) showed that B7-H3 is abundantly expressed in mouse and human adipose tissues, with preferential expression in adipocyte progenitors (APs) [119] (Fig. 5).

Further, they showed that APs lacking B7-H3 experienced a reduction in the glucose and oxidative metabolism. Moreover, white adipocytes derived from B7-H3 silenced adipocyte progenitors showed features of impaired mitochondrial function and increased lipid storage. B7-H3 knock out mice which were on regular chow diet gained significant weight as compared to wild-type controls. These mice developed spontaneous obesity, which was also observed to exhibit diminished regulatory control on metabolic and immune homeostasis. This study shows in addition to its immune regulatory functions, B7-H3 plays an important role in adipocyte progenitor cell differentiation, lipid oxidation, and obesity. The study warrants further research on the role of B7-H3 in this important area.

Moreover, this study reveals a plausible link between diabetes mellitus (DM) and B7-H3. Mice knocked out for B7-H3 showed an increase in tendency toward obesity and associated metabolic syndrome [119]. Therefore, the B7-H3 pathway might be involved in the pathogenesis of type I DM (via immunoregulatory role) and type II DM (obesity and insulin resistance). In this regard, to our knowledge, only one descriptive study reported the association between type I DM and the serum level of B7-H3 [120]. In this study the serum level of B7-H3 in type 1 DM was significantly higher as compared to healthy controls. Given this evidence, the role of B7-H3 in the pathological process in DM should be further explored.

### Effects of post-translational modifications on B7-H3

Post-translational modification of proteins (PTMs) is a covalent modification in protein side chains after translation. PTMs increase the functional diversity of the proteins by the covalent addition of functional groups, proteolytic cleavage of regulatory subunits, or degradation of partial or entire proteins. These modifications include phosphorylation, glycosylation, ubiquitination, nitrosylation, methylation, acetylation, and lipidation and PTMs affect normal cellular functions and may contribute to disease pathogenesis including cancer [121].

PTMs of B7-H3 is an under-researched area. Thus far the most studied PTM for B7-H3 is glycosylation. The functionality of several immune checkpoint molecules has been noted to be modulated by PTMs, including B7-H3 [122]. For example, PD-L1 is heavily glycosylated and this modification is required to interact with its receptor, PD-1, and suppress anti-tumor immunity [123, 124].

B7-H3 is a highly glycosylated protein. In a comprehensive study done by Huang et al., a higher expression of glycosylated B7-H3 is associated with triple-negative breast cancer (TNBC) progression and is a marker of poor patient survival [125]. In addition, they showed that N-glycosylation is required for B7-H3 protein stability and its expression on the surface of cell membrane. Furthermore, N-glycosylation of B7-H3 is required for its immunosuppressive function in TNBC cells. In support of this study, it has been also noted that B7-H3 is aberrantly glycosylated on oral cancer cells [126], although the functional importance of the modification has not been fully elucidated. Other than glycosylation, there are other forms of B7-H3 post-translational modifications (Table 2).

**Table 2:** Types of the post-translational modifications and the modification sites of B7-H3. ( *J. Proteome Res.* 2009, 8, 2, 651–661; Nat Commun (2021) 12(1):2672)

PTMs Residue	PTMS Class	PTM Peptides
N91	N-Glycosylation	ANRTALFPDLLA QGNASL
N104	N-Glycosylation	AQGNASLRL
N189	N-Glycosylation	TGNVTTSQMANEQGLF
N215	N-Glycosylation	RVVLGANGTYSCL
N322	N-Glycosylation	PDLLAQGNASLRLQR
N407	N-Glycosylation	QGVPLTGNVTTSQMA
N433	N-Glycosylation	LRVVLGANGTYSCLV
S513-p	Phosphorylation	QDGEGEGsKtALQPL
T515-p	Phosphorylation	GEGEGsKtALQPLkH
K521-ub	ubiquitination	KtALQPLkHsDskED
K526-ub	ubiquitination	PLkHsDskEDDGQEI

These studies suggest that PTMs of B7-H3 is implicated in the pathogenesis of cancer, and that understanding the molecular mechanisms of modifications and identifications of regulatory factors is warranted. It is also worthwhile to explore the potential impacts of the PTMs on therapeutics targeting B7-H3. To better understand B7-H3’s function and mechanism of actions, more studies should be carried out in this area. Several B7-H3 PTM modification sites have been predicted and Table 2 shows the list of possible PTMs.

### Interaction between B7-H3 and microbiota

Mounting evidence supports that microbiota is an important factor that exerts profound impact on many aspects of cell functions and is involved in the process of many diseases, including cancer [127–129]. Recent reports show that microbiota can affect the expression of B7-H3. *H. pylori* infection elicits B7-H3 expression on gastric epithelial cells through type 4 secretion system (T4SS) components CagA and cell wall peptidoglycan fragment, and the T4SS cell signaling pathway involves modulation of p38MAPK pathway. Th17 cells, Treg cells and a mixed Th1/Th2 response increased during *H. pylori* infection. Human biopsy samples collected from gastritis biopsies and gastric tumors showed an increased B7-H3 and Th2 responses in *H. pylori* strains associated with gastritis [130].

Intriguingly a recent report describes that there is a microbiota-dependent pathway of crosstalk between myeloid cells, T cells, and tumor cells that inhibits CD8^+^ T cell-dependent anti-tumor immunity through the co-inhibitory protein B7-H3. Bacteria sensing by myeloid cells promote calcineurin and NFAT-dependent IL-6 release and NFAT-dependent IL-6 promotes expression of B7-H3 by tumors and it inhibits CD8^+^ T cell-dependent anti-tumor immunity, while B7-H3 blockade elicits protective T cell responses [131]. So far, the interaction between B7-H3 and microbiota is an under-researched but important area, which needs more in-depth studies.

### Identification of B7-H3 Ligand/Receptor and binding partners

Notwithstanding its promise as a drug target, the receptor(s) for B7-H3 have remained unknown. This has become a major obstacle for B7-H3 research and therapeutic application, since it limits our understanding of its biological functions, challenging the development of therapeutics for B7-H3 targeting [5].

Broadly expressed in many tissues and reports of co-inhibition and co-stimulatory role to T cells, B7-H3 has been previously suspected to bind to the triggering receptor expressed on myeloid cells like transcript 2(TREM-like transcript 2, TLT-2) co-stimulating T cell activation [132]. This study showed that overexpression of this molecule renders T cells more responsive to B7-H3 mediated co-stimulation. However, later intensive studies revealed that the human TLT-2 does not bind human B7-H3 and does not serve as a costimulatory receptor for human B7-H3 [133, 134]. Therefore, in these studies, they could not confirm a role of TLT-2 as a B7-H3 receptor.

In attempts to solve the puzzle, Husain et al., (2019) reported the binding of B7-H3 with a coreceptor for several members of the interleukin cytokine family known as Interleukin-20 receptor alpha (IL-20RA) [135]. This group further confirmed the B7-H3/IL20RA interaction by using a recently developed platform for ligand-receptor interactome in HEK293 cell lines [136]. However, the physical association and biological significance of this interaction has not been validated in other experimental systems, especially in vivo models.

Interleukin-20 receptor alpha (IL-20RA), located in chromosomal region 6q23, is a subunit of the IL-20RA/ IL-20RB receptor dimer for IL-10 family members including IL-19, IL-20, IL-22, IL-24 and IL-26 [137]. Studies have shown that IL-20RA is highly expressed in the skin, lung and reproductive organs targeted by the IL-10 family and has been reported to be involved in inflammatory diseases and tissue repair [138]. Studies demonstrated that IL-20RA promotes stemness features and increases the tumor-initiating ability of breast cancer cells via the JAK1-STAT3-SOX2 signaling pathway. Moreover, IL20RA promotes the chemoresistance of breast cancer cells and upregulates the expression of PD-L1 to compromise the activity of anti-cancer immune cells [139]. Furthermore, IL-20RA was highly expressed in the tumor tissue of colorectal cancer (CRC) and related to the advanced stage and poor patient prognosis. Further functional studies showed that knockdown of IL-20RA inhibited the growth and metastasis of CRC [140]. These functions are highly overlapping with those of B7-H3. Therefore, identification of IL-20RA as a possible binding partner for B7-H3 is an intriguing finding that warrants further validation and investigation. In a recent study, another putative binding partner for B7-H3 has been speculated. Angio-associated migratory cell protein (AAMP), ubiquitously expressed in glioma cells, immune cells, and glioma tissue was identified as interaction partner of B7-H3, using bimolecular fluorescence complementation (BiFC) assay, co-immunoprecipitation (co-IP), and functional assays. Knockdown of AAMP reduced specifically the anti-proliferative effect of B7-H3 in Jurkat cells [141]**.**

## Development of B7-H3 targeting drugs for cancer treatment

The introduction of immune check point therapy, which targets the regulatory pathways of T cells in tumor microenvironment, has changed the paradigm of cancer treatment and improved patient survival significantly. Immune checkpoint blockade (e.g., anti-PD-1/ PD-L1 & anti-CTL4) enhances anticancer immune responses.

**Fig. 6:**
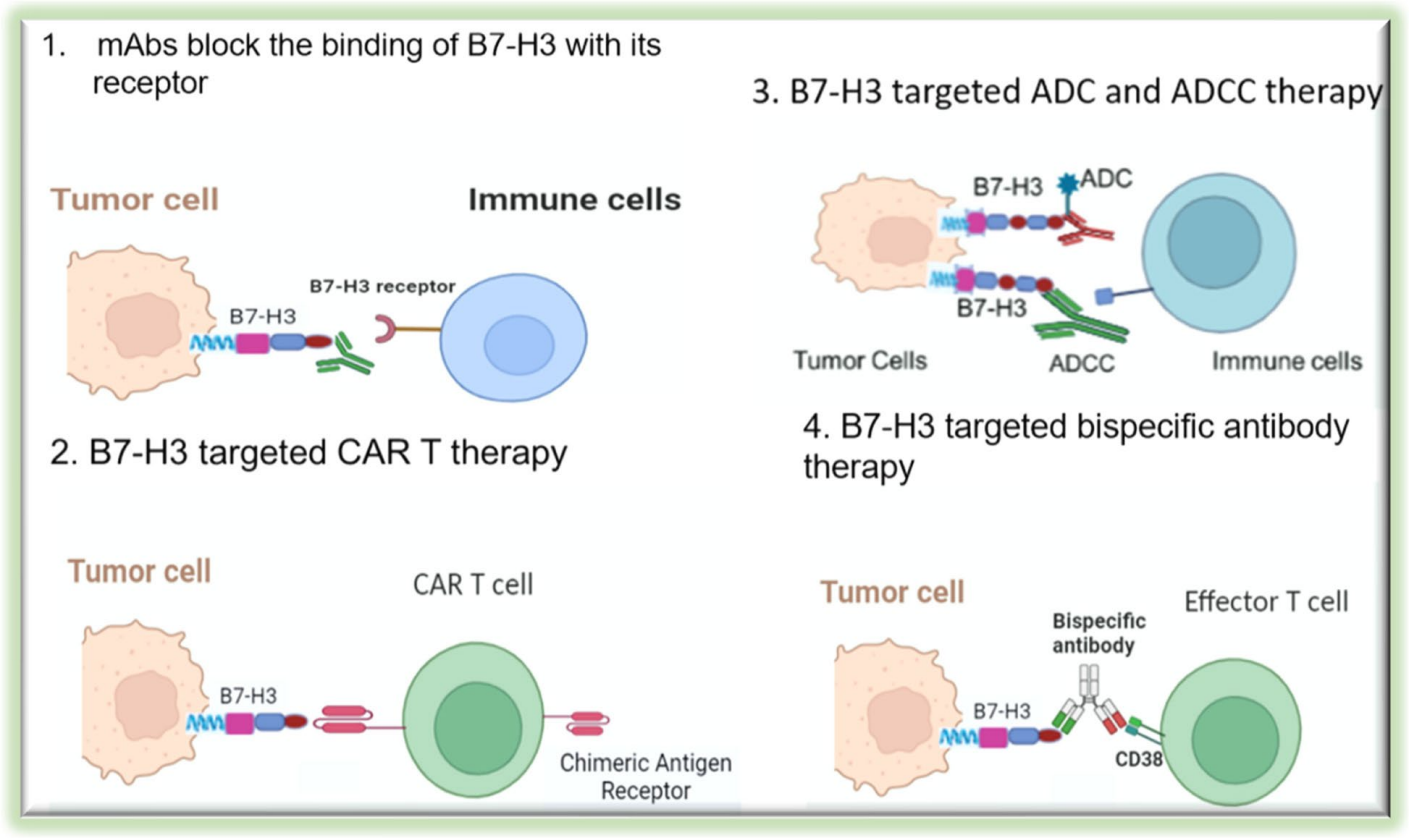
Cancer immunotherapeutic approaches targeting B7-H3. Anti-B7-H3 approaches using different mechanisms including monoclonal antibodies (mAbs), antibody-dependent cell mediated cytotoxicity (ADCC), CAR-T therapy, and Antibody Drug Conjugate (ADC) have been explored

Despite the presence of the overwhelming evidence supporting the tumorigenic effects of B7-H3, there is so far no FDA approved therapeutics/drug targeting B7-H3. A lack of known receptor for B7-H3 makes this molecule difficult to target pharmacologically. It is foreseeable that a discovery of B7-H3 receptor will greatly accelerate the development of effective B7-H3 targeting drug. Nevertheless, due to its importance in cancer pathobiology, anti-B7-H3 approaches using different effector mechanisms including monoclonal antibodies (mAbs), antibody-dependent cell mediated cytotoxicity (ADCC), CAR-T therapy, and Antibody Drug Conjugate (ADC) [142] have been explored (Fig. 6).

Another approach to target B7-H3 expressing tumor cells is targeted radioimmunotherapy (RIT). It is an attractive approach to selectively deliver and target therapeutic radionuclides to both localize and metastatic tumors while sparing normal tissues from the effects of radiation thereby minimizing toxicity [143]. In this regard, as B7-H3 is overexpressed in tumors but restricted in normal tissues, targeting B7-H3 with radionuclides would be particularly advantageous to minimize side effects. Several B7-H3 targeted RIT against preclinical and clinical models have been tested and encouraging results have shown [144, 145]. These studies indicated that targeted delivery of radioisotopes to malignant tissues is another approach to kill cancer cells while sparing normal tissues. B7-H3 targeted RIT combined with immunotherapy or chemotherapy or both would be particularly attractive and worthy of exploring this novel cancer therapeutics.

B7-H3 is a promising target for antibody-based immunotherapy as it is highly expressed on tumor cells, CSCs (critical for tumor metastasis and treatment failure or recurrence), tumor associated vasculature and stroma (involved in angiogenesis) [146]. The expression of B7-H3 and PDL-1, CTLA4 tend to be mutually exclusive. Moreover, the dramatic difference in the B7-H3 expression levels between tumor and normal tissues, provide a large therapeutic window, implying that successful targeting B7-H3 may selectively kill cancer cells and spare normal cells. Several studies have shown promising results using monoclonal antibodies and human-mice chimeric antibodies [147, 148] (Table 3). Furthermore, currently a number of clinical trials are evaluating the therapeutic efficacy of B7-H3 targeting strategies, alone or in combination with other checkpoint inhibitors [146]. There are several review articles extensively discussed targeting B7-H3 in tumor therapy, thus we recommend readers refer to them for additional information [4, 63, 149].

**Table 3:** Summary of selected drugs in testing and clinical trials targeting B7-H3 (ClinicalTrials.gov) and their mechanisms

List of drugs/agents	Mechanisms of Targeting B7-H3	Status	Cancer types	Company/organization
**B7-H3 TriKE(GTB-5550)**	Natural Killer (NK)- cell-based therapy/ADCC	Phase I trial scheduled for 2022	Multiple myeloma	GT BIOPHARMA, Inc
**TAA06 Injection**	B7-H3-targeted CAR-T therapy	FDA approved drug (Orphan drug)	Neuroblastoma	PersonGen BioTherapeutics (Suzhou) Co., Ltd
^**131**^**I-omburtamab**	radiolabeled mAb targeting B7-H3	FDA approval?	Neuroblastoma	Y-mAbs Therapeutics, Inc
**Autologous B7-H3-CAR T**	B7-H3-targeted CAR-T therapy	Phase I	Epithelial Ovarian Cancer	UNC Lineberger Comprehensive Cancer Center
**DS-7300a**	B7-H3 Antibody Drug Conjugate (ADC),	Phase II	Extensive-Stage Small Cell Lung cancer (SCLC)	Daiichi Sankyo, Inc
**MGC018/ MGA012**	Anti-B7-H3 Antibody Drug Conjugate	Phase I/II	Advanced Solid tumors	MacroGenics
**DS-7300a**	anti-B7H3 antibody	Phase I/II	Advanced Solid Malignant Tumors	Daiichi Sankyo Co., Ltd
**B7-H3-CAR T**	B7-H3-targeted CAR-T therapy	Phase I	Recurrent Glioblastoma Multiforme (GBM)	Crystal Mackall, MD, Stanford University
**Neoadjuvant Nivolumab Plus Ipilimumab**	Immunotherapy	Phase 1	Malignant Peripheral Nerve Sheath Tumor	Sidney Kimmel Comprehensive Cancer Center at Johns Hopkins
**177Lu-DTPA-omburtamab**	Radioimmunotherapy (a radioactive labelling of a murine monoclonal antibody targeting B7-H3	Phase I/II	Medulloblastoma	Y-mAbs Therapeutics

## Conclusion and future perspectives

B7-H3 is a transmembrane immunoregulatory protein structurally related to the B7 family of proteins. B7-H3 is overexpressed in cancer cells and primarly localized on the cell membrane. B7-H3 has an inhibitory effect on the activation of T cells and possesses non-immune functions. Despite the lower expression of B7-H3 in the normal tissues, evidence suggest a possible link between B7-H3 and the risk of developing obesity and metabolic disorders such as diabetes mellitus.

Several therapeutic agents have been tested to target B7-H3 and promising results were documented. Because of its tumor promoting functions and its special expression profile, many anti-B7-H3 therapeutic agents are under development by both academia and industry. Unfortunately, B7-H3 research and drug development have been hindered by the lack of understanding of the detailed mechanisms of action and the unknown receptor/functional binding partner. Exploring the receptor and binding partner for B7-H3, its localization in the cell, and the effect of posttranscriptional and posttranslational modifications on its functionality in cancer pathogenesis, its involvement in other diseases, and its function in normal physiology warrants further study to expand our understanding about B7-H3 and develop effective therapeutics.

## Data Availability

Not applicable.

## Abbreviations

ADCC: Antibody-dependent cell mediated cytotoxicity
ADC: Antibody drug conjugate
B7-H3: B7 homolog 3
B7-H4: B7 homolog 4
BRD4: Bromodomain-containing protein 4
CAR-T: Chimeric antigen receptor T cell
CSC: Cancer stem cells
CTL4: Cytotoxic T-lymphocyte-associated protein 4
DM: Diabetes mellitus
GTEx: Genotype-tissue expression
HIF-1α: Hypoxia-inducible factor 1-alpha
HPA: Human protein atlas
IL20RA: Interleukin-20 receptor alpha
MHC: Major histocompatibility complex
miRNA: Micro RNA
PD-1: Programmed cell death protein 1
PD-L1: Programmed cell death ligand 1
PTM: Post-translational modifications
ROS: Reactive oxygen species
TNBC: Triple-negative breast cancer
TCR: T cell receptor
VEGF: Vascular endothelial growth factor
